# Co-Production of NDM-1 and OXA-232 by *Klebsiella pneumoniae*

**DOI:** 10.3201/eid2001.130904

**Published:** 2014-01

**Authors:** Yohei Doi, Jessica A. O’Hara, James F. Lando, Ashley M. Querry, Bethany M. Townsend, Anthony W. Pasculle, Carlene A. Muto

**Affiliations:** University of Pittsburgh School of Medicine, Pittsburgh, Pennsylvania, USA (Y. Doi, J.A. O’Hara, A.M. Querry, B.M. Townsend, A.W. Pasculle, C.A. Muto);; Centers for Disease Control and Prevention, assigned to Allegheny County Health Department, Pittsburgh (J.F. Lando)

**Keywords:** metallo-β-lactamase, carbapenemase, Klebsiella pneumoniae, Escherichia coli, bacteria, New Delhi metallo-β-lactamase, NDM-1, OXA-232, Pittsburgh, Pennsylvania, United States, India

**To the Editor:** New Delhi metallo-β-lactamase 1 (NDM-1) and OXA-48-group β-lactamase have been increasingly reported as carbapenemases responsible for carbapenem resistance in *Enterobacteriaceae* worldwide (*1*). However, in the United States, *Klebsiella pneumoniae* carbapenemase (KPC)–type β-lactamase is the most common carbapenemase among *Enterobacteriaceae*, especially *K*. *pneumoniae*. Isolates producing NDM-1 were first reported in the United States in 2010 (*2*), followed by several case reports and most recently a hospital outbreak in Colorado (*3*–*6*). As for OXA-48-group β-lactamase, 2 cases of infection with OXA-48–producing *K. pneumoniae* were recently reported from Virginia (*7*). We report *K. pneumoniae* co-producing NDM-1 and OXA-232, a variant of OXA-48, and *Escherichia coli* producing NDM-1 that were isolated from the same patient.

A 69-year-old woman was hospitalized in India for subarachnoid hemorrhage in January 2013. Her hospitalization was complicated by unsuccessful coil embolization and subsequent hydrocephalus. A ventriculoperitoneal shunt was inserted, and she was transferred to an acute care hospital in Pittsburgh, Pennsylvania, USA, for further management in February 2013. She underwent reinsertion of the shunt and was discharged to a long-term care facility (LTCF 1). She was readmitted to the same hospital because of fever in March 2013.

A urine culture collected at the time of readmission grew carbapenem-resistant *K. pneumoniae* and extended-spectrum β-lactamase–producing *E. coli*. Although production of KPC-type β-lactamase was initially suspected in *K. pneumoniae*, the unusually high level of resistance to amikacin (MIC >32 μg/mL) and gentamicin (MIC >8 μg/mL) increased concern for presence of an NDM-1 producer, which is frequently highly resistant to aminoglycosides because of production of 16S rRNA methyltransferase (*8*). A modified Hodge test showed a positive result for carbapenemase production, and a metallo-β-lactamase Etest (bioMérieux, Marcy l’Etoile, France) showed a positive result for metallo-β-lactamase production.

PCR and sequencing identified NDM-1 and OXA-232, a 5-aa variant of OXA-48 recently reported in *K. pneumoniae* isolates from India (*9*). Presence of the gene for 16S rRNA methyltransferase (*armA*) was also confirmed by PCR and sequencing and accounted for the high-level aminoglycoside resistance. The isolate belonged to sequence type (ST) 14, as determined by multilocus sequence typing, and has been reported to be common among NDM-1–producing *K. pneumoniae* in Europe (*10*).

The patient was discharged to LTCF 1 but was readmitted because of recurrent fever. A urine culture collected at this admission grew carbapenem-resistant *K. pneumoniae* and carbapenem-resistant *E. coli*. This *E. coli* isolate belonged to ST95 and was positive for the NDM-1 gene but negative for the OXA-48 group and *armA* genes. The original extended-spectrum β-lactamase–producing *E. coli* isolate belonged to ST3865, which is distinct from ST95. Therefore, it is likely that the patient was already colonized by NDM-1–producing *E. coli* ST95 at the time of the first admission, but this colonization was not detected in a clinical culture at that time. All *K. pneumoniae* and *E. coli* isolates remained susceptible to fosfomycin and colistin.

The patient did not receive any antimicrobial drug therapy specific for these isolates because she was deemed to be only colonized with them in the urine. Enhanced contact precautions were also implemented at the time of PCR confirmation of the NDM-1 gene. These precautions included all components of contact precautions (handwashing, gowns, gloves, disinfected/dedicated equipment), and dedicated personnel monitored compliance with these measures around the clock.

The patient was eventually discharged to another long-term care facility (LTCF 2) in April 2013. A point surveillance testing for NDM-1–producing *Enterobacteriaceae* by using rectal swab specimens was conducted for all inpatients at the acute-care hospital and for all residents of the unit at LTCF 2. Testing did not identify any other patients colonized with NDM-1–producing *Enterobacteriaceae*.

In transformation and conjugation experiments, transformants carrying the OXA-232 gene were obtained from *K. pneumoniae*, but those carrying the NDM-1 gene could not be obtained by either method, suggesting that the 2 genes were not located on the same plasmid. For *E. coli*, transformants and transconjugants carrying the NDM-1 gene were obtained, which indicated that this gene was located on a self-conjugative plasmid.

Detection of NDM-1– or OXA-48-group–producing *Enterobacteriaceae*, in particular *K. pneumoniae*, poses a diagnostic challenge in regions to which KPC-producing *K. pneumoniae* is endemic. In our case, recognition of resistance to multiple aminoglycosides by an automated instrument, which was confirmed to be high level by the disk diffusion method (i.e., no inhibition zone), prompted early detection and implementation of appropriate infection prevention measures. Production of 16S rRNA methyltransferase by KPC-producing *K. pneumoniae* is extremely rare, and no cases have been identified in the United States. Therefore, we propose that high-level resistance to amikacin and gentamicin can serve as a clue for suspecting potential NDM-1–producing isolates in clinical diagnostic laboratories.

Conversely, *Enterobacteriaceae* producing OXA-48-group carbapenemase, including variants such as OXA-232, do not have characteristic susceptibility patterns and may easily not be recognized in areas with a high background prevalence of KPC-producing organisms. Therefore, organisms producing OXA-48 or their variants might have already spread in the United States.

## References

[1] Nordmann P, Naas T, Poirel L. Global spread of carbapenemase-producing *Enterobacteriaceae.* Emerg Infect Dis. 2011;17:1791–8 . 10.3201/eid1710.110655 22000347 PMC3310682

[2] Centers for Disease Control and Prevention. Detection of *Enterobacteriaceae* isolates carrying metallo-β-lactamase—United States, 2010. MMWR Morb Mortal Wkly Rep. 2010;59:750 . 20577157

[3] Mochon AB, Garner OB, Hindler JA, Krogstad P, Ward KW, Lewinski MA, New Delhi metallo-β-lactamase (NDM-1)–producing *Klebsiella pneumoniae*: case report and laboratory detection strategies. J Clin Microbiol. 2011;49:1667–70 . 10.1128/JCM.00183-11 21325558 PMC3122842

[4] Savard P, Gopinath R, Zhu W, Kitchel B, Rasheed JK, Tekle T, First NDM-positive *Salmonella* sp. strain identified in the United States. Antimicrob Agents Chemother. 2011;55:5957–8 . 10.1128/AAC.05719-11 21968356 PMC3232776

[5] Centers for Disease Control and Prevention. Carbapenem-resistant *Enterobacteriaceae* containing New Delhi metallo-β-lactamase in two patients—Rhode Island, March 2012. MMWR Morb Mortal Wkly Rep. 2012;61:446–8 . 22717513

[6] Centers for Disease Control and Prevention. Notes from the field: hospital outbreak of carbapenem-resistant *Klebsiella pneumoniae* producing New Delhi metallo-β-lactamase—Denver, Colorado, 2012. MMWR Morb Mortal Wkly Rep. 2013;62:108 . 23407128 PMC4604804

[7] Mathers AJ, Hazen KC, Carroll J, Yeh AJ, Cox HL, Bonomo RA, First clinical cases of OXA-48-producing carbapenem-resistant *Klebsiella pneumoniae* in the United States: the “menace” arrives in the new world. J Clin Microbiol. 2013;51:680–3 . 10.1128/JCM.02580-12 23175248 PMC3553872

[8] Berçot B, Poirel L, Nordmann P. Updated multiplex polymerase chain reaction for detection of 16S rRNA methylases: high prevalence among NDM-1 producers. Diagn Microbiol Infect Dis. 2011;71:442–5 . 10.1016/j.diagmicrobio.2011.08.016 22000158

[9] Potron A, Rondinaud E, Poirel L, Belmonte O, Boyer S, Camiade S, Genetic and biochemical characterisation of OXA-232, a carbapenem-hydrolysing class D β-lactamase from *Enterobacteriaceae.* Int J Antimicrob Agents. 2013;41:325–9 . 10.1016/j.ijantimicag.2012.11.007 23305656

[10] Giske CG, Froding I, Hasan CM, Turlej-Rogacka A, Toleman M, Livermore D, Diverse sequence types of *Klebsiella pneumoniae* contribute to the dissemination of *bla*_NDM-1_ in India, Sweden, and the United Kingdom. Antimicrob Agents Chemother. 2012;56:2735–8 . 10.1128/AAC.06142-11 22354295 PMC3346639

